# Integrin α5β1-Ang1/Tie2 receptor cross-talk regulates brain endothelial cell responses following cerebral ischemia

**DOI:** 10.1038/s12276-018-0145-7

**Published:** 2018-09-05

**Authors:** Defang Pang, Lu Wang, Jing Dong, Xiaoyin Lai, Qijuan Huang, Richard Milner, Longxuan Li

**Affiliations:** 1 Department of Special Outpatient Service, Gongli Hospital, The Second Military Medical University, Shanghai, 200135 P. R. China; 2 Department of Neurology, Gongli Hospital, The Second Military Medical University, Shanghai, 200135 P. R. China; 3 The Graduate School, Ningxia Medical University, Yinchuan, Ningxia 750004 P. R. China; 4 Department of Pharmacy, Gongli Hospital, The Second Military Medical University, Shanghai, 200135 P. R. China; 5 Department of Neurology, Taishan People’s Hospital, Taishan, 529200 P. R. China; 6 Department of Molecular Medicine, The Scripps Research Institute, 10550 North Torrey Pines Road, La Jolla, CA 92037 USA

## Abstract

We have previously demonstrated that in response to cerebral ischemia (CI), the growth factor angiopoietin-1 (Ang1) and α5β1 integrin are both induced in cerebral vessels, which likely provide positive signals driving the endogenous angiogenic response and vascular protection after CI. However, the precise relationship between endothelial Ang1 and α5β1 integrin after CI remains poorly understood. Here, we investigated the effects of the interaction between the Ang1/Tie2 system and α5β1 integrin on brain endothelial cells (BECs) under cerebral ischemic conditions in vivo and in vitro. Immunofluorescence analysis demonstrated that integrin α5β1 co-localized with Tie2/phosphorylated Tie2 on cerebral vessels in the penumbra. The in vitro study showed that oxygen–glucose deprivation/restoration (OGD/R) induced the expression of the Ang1 receptor Tie2 on BECs in a manner similar to that for integrin α5 and Ang1 in response to OGD/R, accompanied by increased activation of Tie2 and its downstream effectors focal adhesion kinase (FAK) and Akt. Knockdown of α5 integrin markedly suppressed OGD/R-induced Tie2 receptor activation in BECs, while in contrast, priming BECs with Ang1 promoted the expression of α5 integrin as well as the Tie2 downstream transcription factor Ets-1 in OGD-treated BECs. In line with this, Ets-1 knockdown significantly attenuated Ang1-mediated upregulation of α5 integrin. Functionally, Ang1 induced cell migration and tube formation of BECs after OGD, but this effect was inhibited by diminishment of the levels of α5 integrin in BECs. Taken together, our data indicate that the Ang1/Tie2 system cross-talks with integrin α5β1 in BECs after CI, which may contribute to the endogenous angiogenic vascular protective response following CI.

## Stroke: Interacting signals promote new blood vessel formation

Interactions between two molecular signaling systems on the surface of brain blood vessel cells may regulate the response to cerebral ischemic (CI) strokes, in which the blood supply and hence oxygen to part of the brain is interrupted by blockages, such as blood clots. Researchers led by Longxuan Li at Gongli Hospital, the Second Military Medical University, Shanghai, and Richard Milner at the Scripps Research Institute, La Jolla, USA, used mice and cultured cells to study these interactions. The results suggest that the cell surface receptor proteins that mediate both signaling systems localize in the same area of blood cell membranes in response to a CI stroke. Molecular “cross-talk” between these systems may promote the formation of new blood vessels to repair the damage caused by a stroke. These insights could assist development of drugs to treat CI strokes.

## Introduction

Angiogenic remodeling has been described in the penumbral regions in animal models of stroke as well as in the ischemic brains of stroke patients^1–4^. Greater microvessel density in the ischemic border has been reported to correlate with longer survival in stroke patients^4^. It is commonly believed that angiogenic remodeling may enhance cerebral perfusion, which leads to improved functional recovery after stroke^5^.

Angiopoietin-1 (Ang1) is an endogenous ligand for the vascular endothelial receptor tyrosine kinase Tie2. Mounting evidence shows that activation of Tie2 signaling pathways by Ang1 promotes endothelial cell/endothelial progenitor cell migration and sprouting, and protects the endothelial cells from apoptosis by phosphorylating the downstream effectors focal adhesion kinase (FAK) and Akt, a serine/threonine-specific protein kinase^6–8^. Therefore, the Ang1/Tie2 system is considered as a promising molecular target for promoting therapeutic neovascularization and vascular protection.

In a recent study, we observed that in response to cerebral ischemia, Ang1 and the pro-angiogenic factor α5β1 integrin were both induced in cerebral vessels and brain endothelial cells (BECs) in vivo and in vitro^1,9^. Moreover, extensive co-localization of Ang1 with α5 integrin was found on angiogenic blood vessels in the ischemic penumbra^9^. Interestingly, BEC proliferation and tight junction protein expression followed the same time course as the expression of α5β1 and Ang1, suggesting a close correlation between ɑ5 integrin and Ang1 in promoting angiogenesis and tight junction formation in the blood–brain barrier (BBB) after ischemic stroke.

Tie2/α5β1 interactions modulate Ang-1-triggered signaling pathways in endothelial cells^10^. There is also evidence that stimulation of peripheral blood stem cells enriched by granulocyte colony-stimulating factor mobilization and apheresis (mobPBSCs) with cartilage oligomeric matrix protein (COMP)–Ang1 (a soluble, stable and potent Ang1 variant) induces the expression of α5β1 integrin^11^. However, the precise relationship between the Ang1/Tie2 system and α5β1 integrin after cerebral ischemic stroke is poorly understood. The aim of this study was to investigate the cross-talk between the pro-angiogenic factor α5β1 integrin and Ang1 in BECs and the functional implications of this interaction in mediating BEC migration and tube formation under ischemic conditions in vivo and in vitro.

## Materials and methods

### Experimental animals

Male C57Bl/6 mice weighing 20–25 g at the time of surgery were used for all experiments. The present study was conducted in accordance with the NIH guidelines for the care and use of animals in research and under protocols approved by the Animal Care and Use Committee of Gongli Hospital, Pudong New Area, Shanghai.

### MCAO model

Focal cerebral ischemia/ischemic stroke was induced by reversible right middle cerebral artery occlusion (MCAO) surgery under pentobarbital anesthesia, followed by reperfusion as described previously^9^. After 90 min of MCAO, the mice were briefly re-anesthetized and reperfusion was commenced. Sham animals were subjected to an identical procedure, but they did not undergo MCAO. Intra-ischemic neurological deficit was confirmed and scored as previously described ^2^.

### Cell culture

Immortalized mouse BECs of the bEnd3 cell line were obtained from the Shanghai Bioleaf Biotech Co., Ltd. Primary human brain microvascular endothelial cells (HBMECs) were purchased from Cell Systems (Kirkland, WA, USA). Cells (bEnd3 and HBMECs) were grown in six-well plates pre-coated with type I or IV collagen (10 μg/mL, Sigma, for 2 h at 37 °C). The culture medium was endothelial basal medium (EBM-2) (Lonza, CC-3156) supplemented with 10% FBS (Gibco), ascorbic acid, L-glutamine, penicillin/streptomycin, and human basic fibroblast growth factor (bFGF) (all from Sigma). Cells were maintained in a humidified incubator at 37 °C and 5% CO2, and the medium was changed every 48 h.

### Oxygen–glucose deprivation and restoration (OGD/R)

BEC cultures were subjected to ischemia-like injury through oxygen–glucose deprivation (OGD) for 4 h by placing the cultures in a deoxygenated glucose-free balanced salt solution (BSS0) in an anaerobic chamber (Forma, Thermo Scientific, Asheville, NC, USA) with an atmosphere of 5% CO_2_ and 95% N_2_. After 4 h of OGD, the cultures were returned to control conditions (restoration) by adding 5.5 mM glucose to the media under normoxic conditions. Control cultures (no injury) were incubated with a balanced salt solution containing 5.5 mM glucose (BSS5.5). All cultures were maintained in a humidified 37 °C incubator.

### Immunofluorescent studies and antibodies

Mice at different time points of reperfusion were euthanized by perfusion with ice-cold saline, and then, the brains were rapidly dissected and stored at −80 °C. Immunofluorescence (IF) studies were performed as previously described^9^ on 10-mm-thick frozen coronal sections. The PE-conjugated rat anti-mouse α5 (CD49e) (clone 5H10-27, 1:150) antibody was purchased from BD Pharmingen (La Jolla, CA, USA), phospho-Tie2 (Tyr1108) polyclonal antibody (PA5-38339, 1:100) was obtained from Thermo Fisher Scientific (Rockford, IL, USA), and mouse anti-Tie2 antibody (AB24859, 1:25) was obtained from Abcam (Cambridge, MA, USA). The Cy3-conjugated goat anti-rat secondary antibody was obtained from EarthOx. AlexaFluor 488-conjugated goat anti-mouse and rabbit secondary antibodies were purchased from Jackson Immunoresearch (West Grove, PA, USA). The negative controls for staining and confocal imaging were used to confirm the coexistence of vessel proteins.

### siRNA transfection

GIPZ Lentiviral mouse Itgα5 (Cat# VGH5526-EG16402), Ets-1 (Cat# VGH5526-EG23871), small hairpin RNA (shRNA), and non-targeting shRNA controls were purchased from Dharmacon (Lafayette, CO, USA). BECs, at a concentration of 2.4 × 10^5^, were plated in six-well plates in 2 mL of growth medium for 24 h prior to transfection. The following day, transfection was performed when the cells reached 70–80% confluence. shRNAs, including GIPZ Itga5, Ets-1, or non-silencing shRNA viral particle, were incubated with DharmaFECT kb transfection reagent (Lafayette, CO, USA), according to the manufacturer’s reverse transfection protocol. After the medium was replaced with a new growth medium, transfection complexes were added to each well (final concentration of 2 µg/mL per well) and incubated at 37 °C in a CO2 incubator. Forty-eight hours after transfection, cells were subjected to OGD/R treatment with or without drug administration. Silencing of specific genes was confirmed by western blot.

### Drug administration

After 4 h OGD, BECs were transferred to normal conditions to terminate the OGD and begin restoration. To detect the Ang1 effects on BEC α5 integrin expression/α5β1 effects on BEC Tie2 signaling after OGD, control or shRNA Ets-1/shRNA α5-treated BECs were stimulated with or without Ang1 (50, 100, or 200 ng/mL), as indicated for 15 min, 1 h or 12 h at the beginning of restoration. Cells were then harvested for western blotting at different time points after restoration initiation.

### Western blot analysis

BECs were harvested and lysed with lysis buffer (1% NP-40, 50 mM TrisHCl, pH 8.0, 150 mM sodium chloride) supplemented with protease and phosphatase inhibitor cocktails. Protein concentration was determined using the BCA protein assay kit (Eppendorf-Bio photometer, Germany). Western blotting and semi-quantitative analysis were performed as described previously^9^. The primary antibodies used were rabbit anti-angiopoietin-1 (AB10516, 1:1000, Merck Millipore, Darmstadt, Germany), rabbit anti-α5 (AB1928, 1:1000, Merck Millipore, Darmstadt, Germany), rabbit anti-α5 (#4705, 1:1000, Cell Signaling Technology, Inc., Danvers, MA, USA), mouse anti-Ets-1 (sc-55581, 1:200, Santa Cruz Biotechnology, INC., USA), rabbit anti-phospho-Tie-2 (Y992) (AF2720, 1:400, R&D Systems, Inc. Minneapolis, MN, USA), mouse anti-Tie2 (ab24859, 1:100, Abcam, Cambridge, MA, USA), rabbit anti-phospho-FAK (Tyr397) (ABT135,1:1000, EMD Millipore Corporation, Temecula, CA, USA), rabbit anti- FAK (06-543, EMD Millipore Corporation, Billerica, Massachusetts, USA 1:200), rabbit anti-phospho-Akt (Ser473) (9271 L, Cell Signaling Technology, Danvers, Massachusetts, USA 1:1000), rabbit anti-Akt (9272 s, 1:1000, Cell Signaling Technology, Danvers, Massachusetts, USA), and β-actin (1:1000, Neomarker, Fremont, CA). For each sample, the protein levels were first normalized to the level of β-actin and then expressed as the fold increase over the level of the NO-OGD/R control group.

### Scratch wound migration assay

BECs were transfected with the indicated shRNA, then seeded in six-well tissue culture plates (5 × 10^5^ cells per well), and cultured to confluence. The confluent monolayer cells were subjected to OGD for 4 h and then scratched using a sterile 200-μL pipette tip. The remaining wounded monolayer was washed twice with PBS to clear the cell debris. The BECs were re-fed with mitomycin C (1 mM, Sigma-Aldrich) containing serum-free EBM-2 or serum-free EBM-2 containing Ang1 (100 ng/mL) and then left to heal for 24 h at normoxia. Photographs were taken at the same site at 0 and 24 h after the injury. The healing of the wounds was assessed by measuring the wound gap. The experiments were undertaken in triplicate and were blindly analyzed.

### Tube formation assay

BECs were transfected with the indicated shRNA and then exposed to OGD for 4 h. The in vitro tube formation assay was carried out as previously described^12,13^. In brief, 250 μL of growth factor-reduced Matrigel (BD Biosciences, San Diego, CA) was transferred to a well of a 24-well tissue culture plate and polymerized for 30 min at 37 ℃. BECs (1 × 10^5^ cells/well) were plated onto the layer of Matrigel in the presence or absence of Ang1 (100 ng/mL) as indicated. Twelve hours later, four representative fields were examined, and the average total areas of complete tubes formed by cells per unit area were compared. This examination was repeated three times and was blindly analyzed.

### Statistical analysis

All quantified data represent the mean ± SEM. Statistical significance was assessed by the *t* test and one- or two-way analysis of variance (ANOVA), and a Bonferroni post-hoc test was used to test multiple comparisons. All statistical analyses were performed with SPSS (version 16.0; SPSS, Chicago, IL, USA) and significance was defined as *P* < 0.05.

## Results

### The influence of OGD/R on the temporal expression pattern of Tie2 and the activation of downstream signaling molecules in BECs

In a recent study, we demonstrated that OGD/R induced a markedly increased expression of both integrin α5 and Ang1 on BECs, with maximum levels of expression reached 48–72 h post-restoration^9^. In the current study, we wanted to evaluate whether OGD/R also influenced BEC expression of the Ang1 receptor Tie2. To examine this, we subjected bEnd3 cells and HBMECs to 4 h of OGD, followed by up to 72 h of restoration (normal glucose and normoxia). We then examined total protein, the phosphorylated form of Tie2, and the expression of integrin α5 and Ang1 by western blotting.

**Fig. 1:**
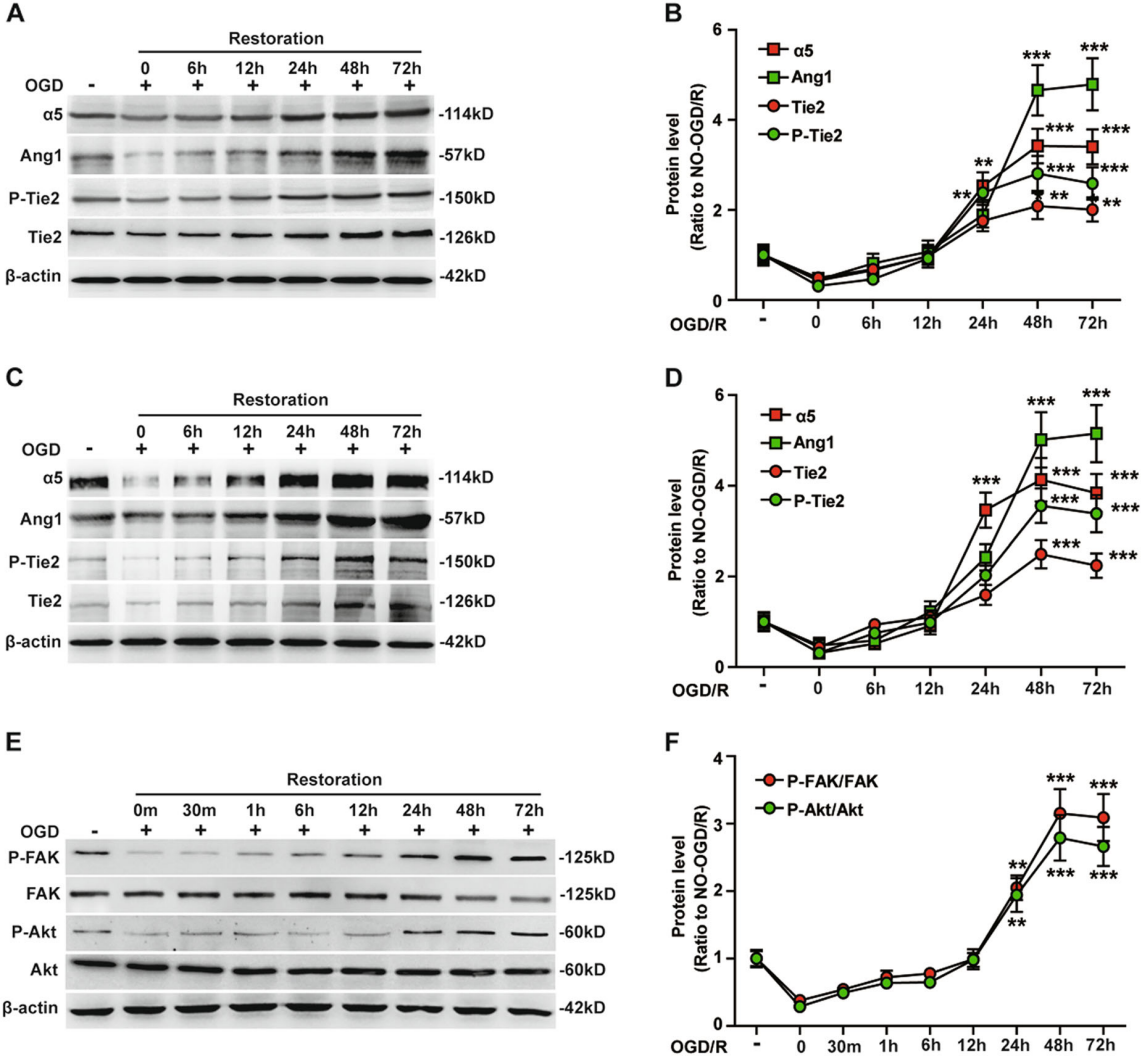
OGD/R induces the upregulation of Tie2 expression and activation of the downstream signaling molecules FAK and Akt in BECs. BECs (bEnd3 cells and HBMECs) were subjected to 4 h of oxygen–glucose deprivation (OGD) followed by 72 h of restoration (R) at 37 °C. Cell lysates at each time point were analyzed by western blotting using antibodies specific for integrin α5, Ang1, and total protein or phosphorylated form of Tie2, focal adhesion kinase (FAK) and Akt. **a** Representative western blots of integrin α5, Ang1 and total and phosphorylated form of Tie2 in bEnd3 cells are shown. **b** Protein levels for **A** were quantified by densitometry and are presented as ratios to β-actin. **c** Representative western blots of integrin α5, Ang1 and total and phosphorylated form of Tie2 in HBMECs are shown. **d** Protein levels for C were quantified by densitometry and are presented as ratios to β-actin. **e** Representative western blots of phosphorylated FAK, phosphorylated Akt, total FAK and total Akt are shown. **f** Protein levels were quantified by densitometry and normalized to total FAK/Akt. The NO-OGD/R cells served as a control. Note that the levels of phosphorylated and total Tie2 decreased immediately after OGD insult but then began to increase and continued to increase with time of restoration, reaching a maximum at 48-72 h after restoration, similar to the expression of α5 integrin and Ang1. In the first 12 h following ischemic insult, OGD/R reduced phosphorylation of both FAK and Akt, but by the 24–72 h time point, the levels of both phosphorylated FAK and Akt were significantly elevated compared to pre-OGD conditions, which correlated with the changes of total and phosphorylated form of Tie2. Data represent the mean ± SEM and were analyzed by one-way ANOVA **(*n*** = 5). **P < 0.01, ***P < 0.001 compared with NO-OGD/R control

As shown in Fig. 1a, b, western blot analysis revealed that compared to control conditions, the levels of total and phosphorylated Tie2 decreased immediately after OGD insult in bEnd3 cells, but then began and continued to increase with time of restoration to reach a maximum between 48 and 72 h after restoration. BEC expression of α5 integrin and Ang1 (Fig. 1a, b, additional Fig. 1) showed a similar response.

Quantification revealed that compared to NO-OGD/R control conditions, the expression of Tie2 was increased 2.38 ± 0.27-fold for phosphorylated Tie2 (*P* < 0.01) and 1.76 ± 0.23-fold for total Tie2 (*P* > 0.05) at 24 h, further increased 2.81 ± 0.39-fold for phosphorylated Tie2 (*P* < 0.001) and 2.09 ± 0.29-fold for total Tie2 (*P* < 0.01) at 48 h, and increased 2.59 ± 0.36-fold for phosphorylated Tie2 (*P* < 0.001) and 2.01 ± 0.27-fold for total Tie2 (*P* < 0.01) at 72 h. Similarly, the expression of α5 integrin was increased 2.53 ± 0.31-fold (*P* < 0.01) at 24 h, further increased 3.42 ± 0.38-fold (*P* < 0.001) at 48 h and increased 3.4 ± 0.39-fold (*P* < 0.001) at 72 h. In parallel, the expression levels of Ang1 were increased 1.89 ± 0.28-fold (*P* > 0.05) at 24 h, significantly increased 4.66 ± 0.56-fold (*P* < 0.001) at 48 h, and further increased 4.79 ± 0.58-fold (*P* < 0.001) at 72 h (Fig. 1b).

In addition, we used HBMECs to repeat the above studies and found that integrin α5, Ang1, and the total and phosphorylated form of Tie2 in HBMECs changed in similar manners (Fig. 1c, d).

Autophosphorylation of Tie2 triggered by Ang1 binding has been reported to activate specific intracellular signal molecules, including FAK, Akt, and mitogen-activated protein kinase (MAPK) (ERK1/2) in endothelial cells, which have been shown to selectively regulate vascular quiescence and angiogenesis^12,14^. Considering this, we next examined how OGD/R affects phosphorylation of the Tie2 receptor downstream signaling molecules FAK and Akt on BECs.

As expected, in the first 12 h following the ischemic insult, OGD/R induced a reduction in phosphorylation of both FAK and Akt in BECs, but by the 24–48-h time point, the endothelial levels of both phosphorylated FAK and Akt were significantly elevated compared to pre-OGD conditions (Fig. 1e, f), and this was associated with enhanced expression of phosphorylated and total Tie2. Quantification revealed that compared to the NO-OGD/R control conditions, phosphorylation of FAK was increased to 2.05 ± 0.18-fold (*P* < 0.01) at 24 h, further increased to 3.15 ± 0.36-fold (*P* < 0.001) at 48 h, and increased to 3.09 ± 0.35-fold (*P* < 0.001) at 72 h, and the phosphorylation level of Akt was increased to 1.94 ± 0.25-fold (*P* < 0.01) at 24 h, further increased to 2.79 ± 0.34-fold (*P* < 0.001) at 48 h, and increased to 2.66 ± 0.29-fold (*P* < 0.001) at 72 h (Fig. 1f).

### OGD/R-induced Tie2 receptor activation on BECs is mediated by α5β1 integrin

**Fig. 2:**
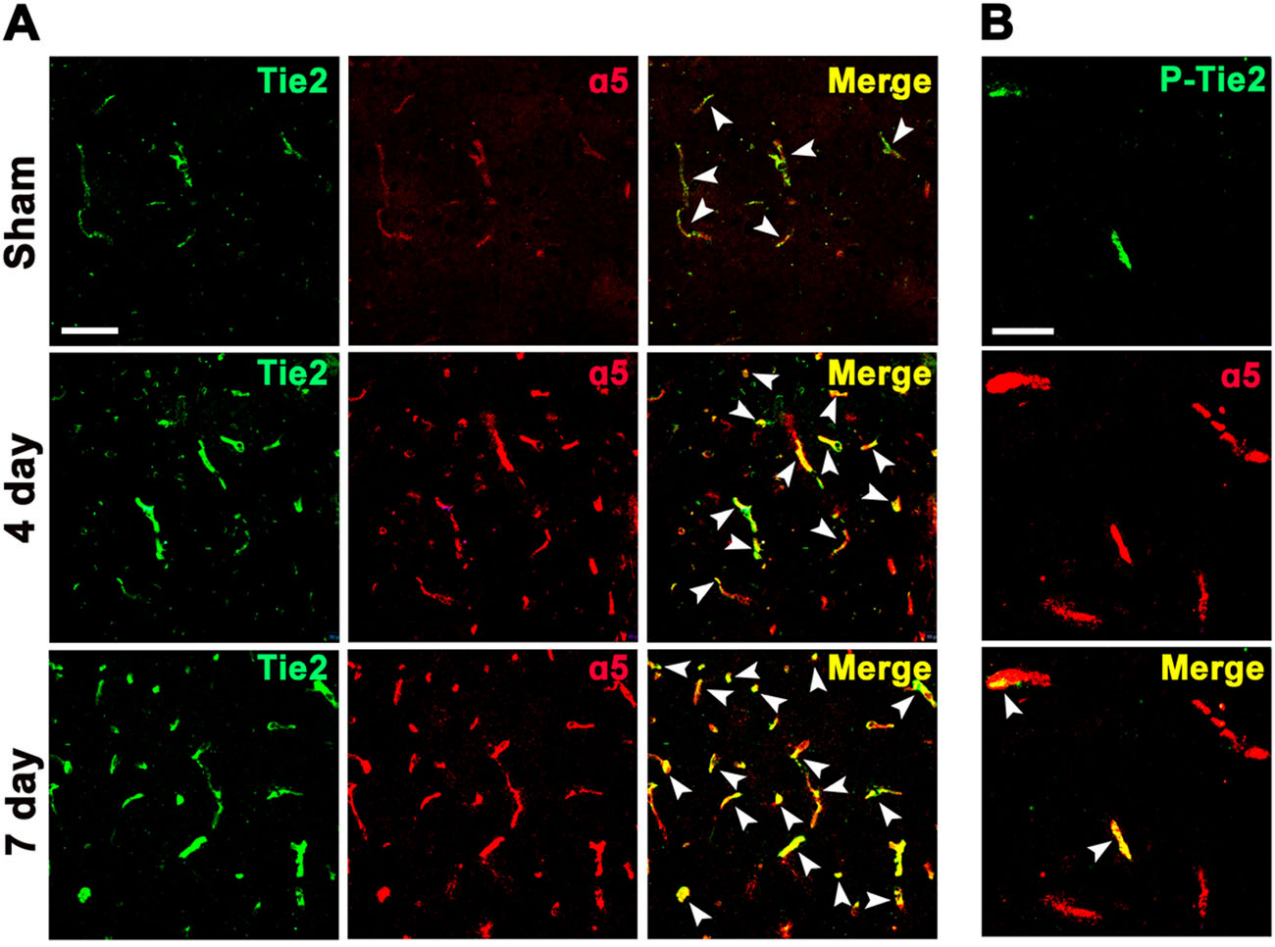
Coexpression of Tie2/P-Tie2 with a5 integrin on cerebral vessels after cerebral ischemia. Dual-IF was performed on frozen sections of ischemic penumbra taken from sham-operated (control) mice at 4 and 7 days after focal cerebral ischemia, using antibodies specific for ɑ5 (Cy-3), Tie2 (AlexaFluor-488) (**a**)/ɑ5 (Cy-3), or P-Tie2 (AlexaFluor-488) (**b**). Scale bar = 50 mm for all panels in figure A, Scale bar = 30 mm for all panels in figure B. Note that cerebral ischemia induced a strong increase in vascular expression of Tie2 and ɑ5 integrin. In the ischemic penumbra, Tie2 and ɑ5 integrin co-localized (white arrows) extensively. In contrast, only relatively low levels of Tie2 co-localize with ɑ5 integrin (white arrows) in the control (sham) brain. The activated Tie2 (P-Tie2) co-localized with ɑ5 integrin (white arrows) in the ischemic penumbra

As Tie2 has been shown to interact selectively and constitutively with α5β1 integrin^10^, we next performed dual-IF to examine whether Tie2/P-Tie2 and α5 integrin subunits show any overlap in their expression profiles after cerebral ischemia. As shown in Fig. 2a, cerebral ischemia induced a strong increase in vascular expression of Tie2 and α5 integrin at days 4 and 7 post ischemia compared with the control (sham) brain. Integrin α5β1 extensively co-localized with Tie2 on cerebral vessels in the ischemic penumbra at 4 and 7 days post ischemia. In addition, the coexpression of P-Tie2 with α5 integrin can be observed on cerebral vessels in the ischemic penumbra 4 days post ischemia (Fig. 2b). These results show that there is a direct interaction between α5β1 integrin and Tie2, and that α5β1 integrin might be involved in the activation of Tie2 after cerebral ischemia.

**Fig. 3:**
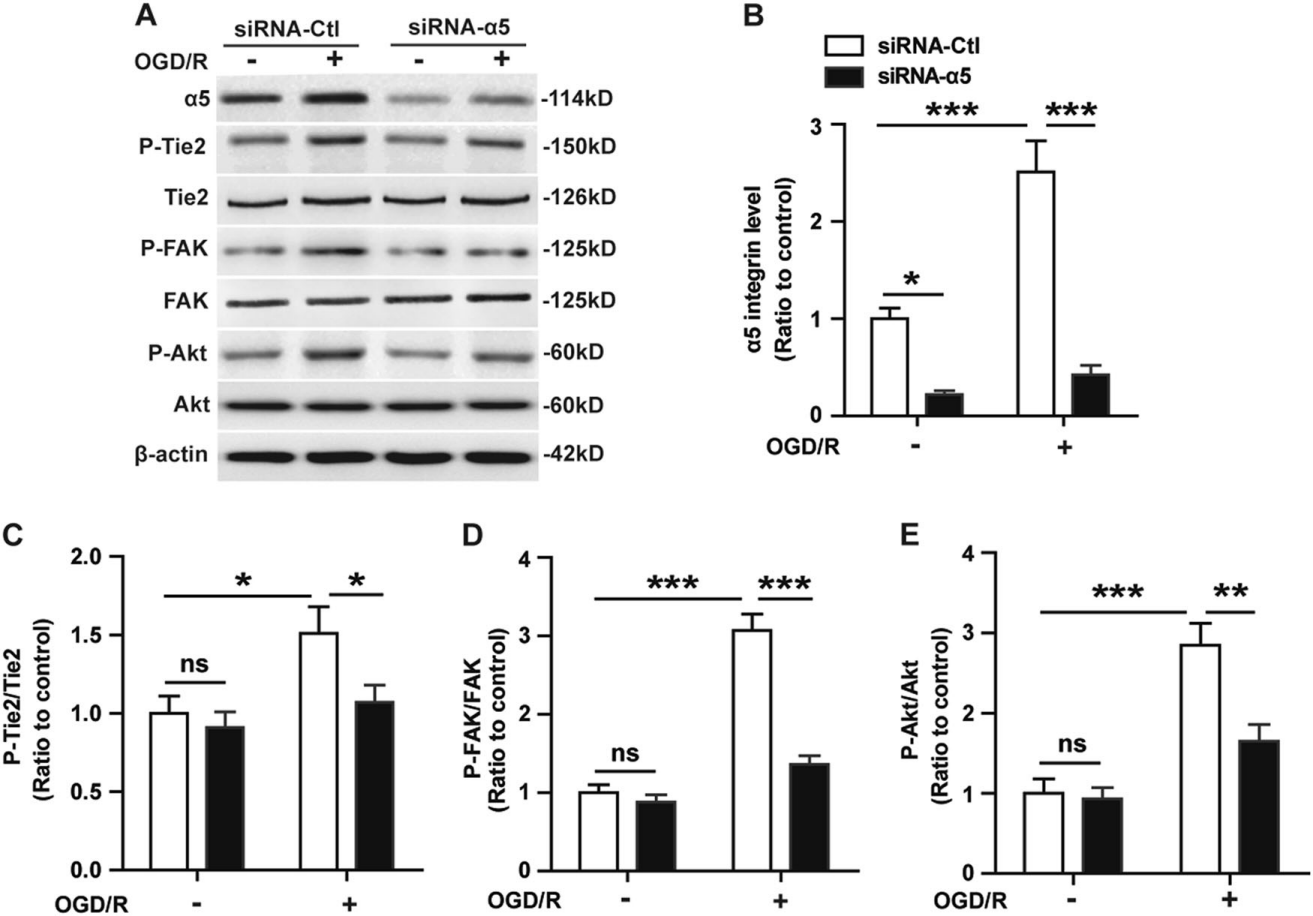
Knockdown of α5 integrin attenuates Tie2 activation in BECs induced by OGD/R. BECs were transfected with the negative control siRNA (siRNA-Ctl) or α5 integrin-specific siRNA (siRNA-α5) for 48 h and then subjected to either 4 h of OGD followed by 48 h of restoration or NO-OGD/R at 37 °C. **a** Cell lysate protein levels were analyzed by western blotting using antibodies specific to integrin α5 and total or phosphorylated forms of Tie2, FAK, and Akt. **b–e**. Bar graphs show the quantitative analyses of western blots as ratios of integrin α5/β-actin (**b**), phosphorylated Tie2/total Tie2 (**c**), phosphorylated FAK/total FAK (**d**), and phosphorylated Akt/total Akt (**e**). The NO-OGD/R siRNA-Ctl-treated cells served as the control. Data represent the mean ± SEM and were analyzed by two-way ANOVA (***n*** = **5**). Note that OGD/R significantly increased the levels of Tie2, FAK, and Akt phosphorylation on siRNA-Ctl-treated BECs relative to the NO-OGD/R siRNA-Ctl-treated controls, but this effect was significantly reduced by diminishing the levels of integrin α5 in the BECs. *P < 0.05, **P < 0.01, ***P < 0.001; ns, not significant

To confirm if α5β1 integrin mediates OGD/R-induced Tie2 activation in BECs, we employed α5 integrin-specific siRNA to knockdown the expression of α5 integrin in BECs and examined the impact of this on Tie2 receptor signaling activation in response to OGD/R. As shown in Fig. 3a–e, 48 h after restoration, expression of phosphorylated Tie2 and its downstream effectors FAK and Akt in the negative control siRNA (siRNA-Ctl)-treated BECs was significantly induced in response to OGD/R relative to the NO-OGD/R siRNA-Ctl-treated cells (the controls) (*P < 0.05 or ***P < 0.001), but this effect was markedly attenuated following integrin α5 knockdown. These data suggest that integrin α5β1 regulates the OGD/R-induced Tie2 receptor activation on BECs.

**Fig. 4:**
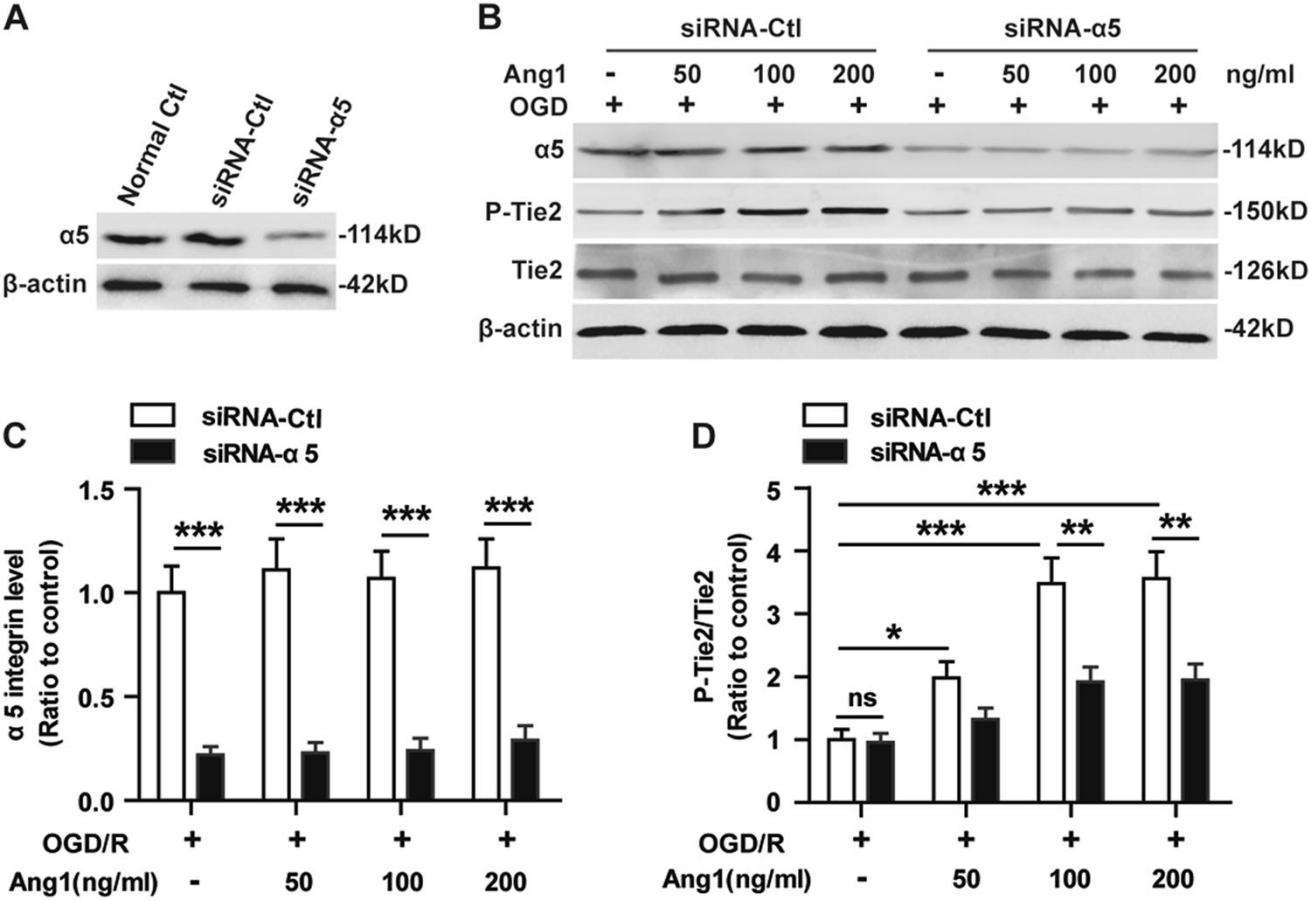
Integrin α5β1 acts to enhance Ang1-activated Tie2 signaling after OGD. **a** BECs were transfected with negative control siRNA (siRNA-Ctl) or α5 integrin-specific siRNA (siRNA-α5) for 48 h. Levels of α5β1 protein were monitored in the control and silenced cells via western blotting. **b–d** BECs were transfected with siRNA-Ctl or siRNA-α5 for 48 h, then subjected to 4 h of OGD, and further stimulated or not with Ang1 (50, 100, or 200 ng/mL) for 15 min under normoxia at 37 °C. **b** The protein levels from OGD-treated BEC lysates were analyzed by western blotting using antibodies specific to integrin α5 and total or phosphorylated forms of Tie2. **c–d** Densitometric analysis shows the relative amount of integrin α5 (**c**) and phosphorylated Tie2/total Tie2 (**d**). The OGD/R siRNA-Ctl-treated cells served as the control. Data represent the mean ± SEM and were analyzed by two-way ANOVA (n = 5). Note that integrin α5 knockdown in BECs significantly decreased α5β1 protein expression after OGD. Knockdown of integrin α5 in BECs did not significantly decrease the levels of Tie2 phosphorylation after OGD. Ang1 significantly increased the levels of Tie2 phosphorylation in a dose-dependent manner on siRNA-Ctl-treated BECs after OGD, but this effect was significantly reduced by integrin α5 knockdown. *P *<* 0.05, ***P < 0.001, ns, not significant

To investigate whether α5β1 integrin directly induces Tie2 signaling, BECs were transfected with negative control siRNA (siRNA-Ctl) or α5 integrin-specific siRNA (siRNA-α5) for 48 h, then subjected to 4 h of OGD, and further stimulated or not with Ang1 (50, 100, or 200 ng/mL) for 15 min under normoxia at 37 °C. As shown in Fig. 4a–d, knockdown of integrin α5 in BECs did not significantly decrease the levels of Tie2 phosphorylation after OGD (Fig. 4b, d). However, Ang1 (at doses of 50, 100 and 200 ng/mL) significantly increased the levels of Tie2 phosphorylation in a dose-dependent manner on siRNA-Ctl-treated BECs after OGD compared with the non-Ang1 treatment group (*P < 0.05 or ***P < 0.001), but this effect was significantly reduced by integrin α5 knockdown (Fig. 4b, d). These data suggest that α5β1 integrin does not directly induce Tie2 signaling, but acts to enhance Ang-1-activated Tie2 signaling on BECs after OGD.

### Priming of BECs with Ang1 promotes the expression of α5 integrin and Ets-1 after OGD

**Fig. 5:**
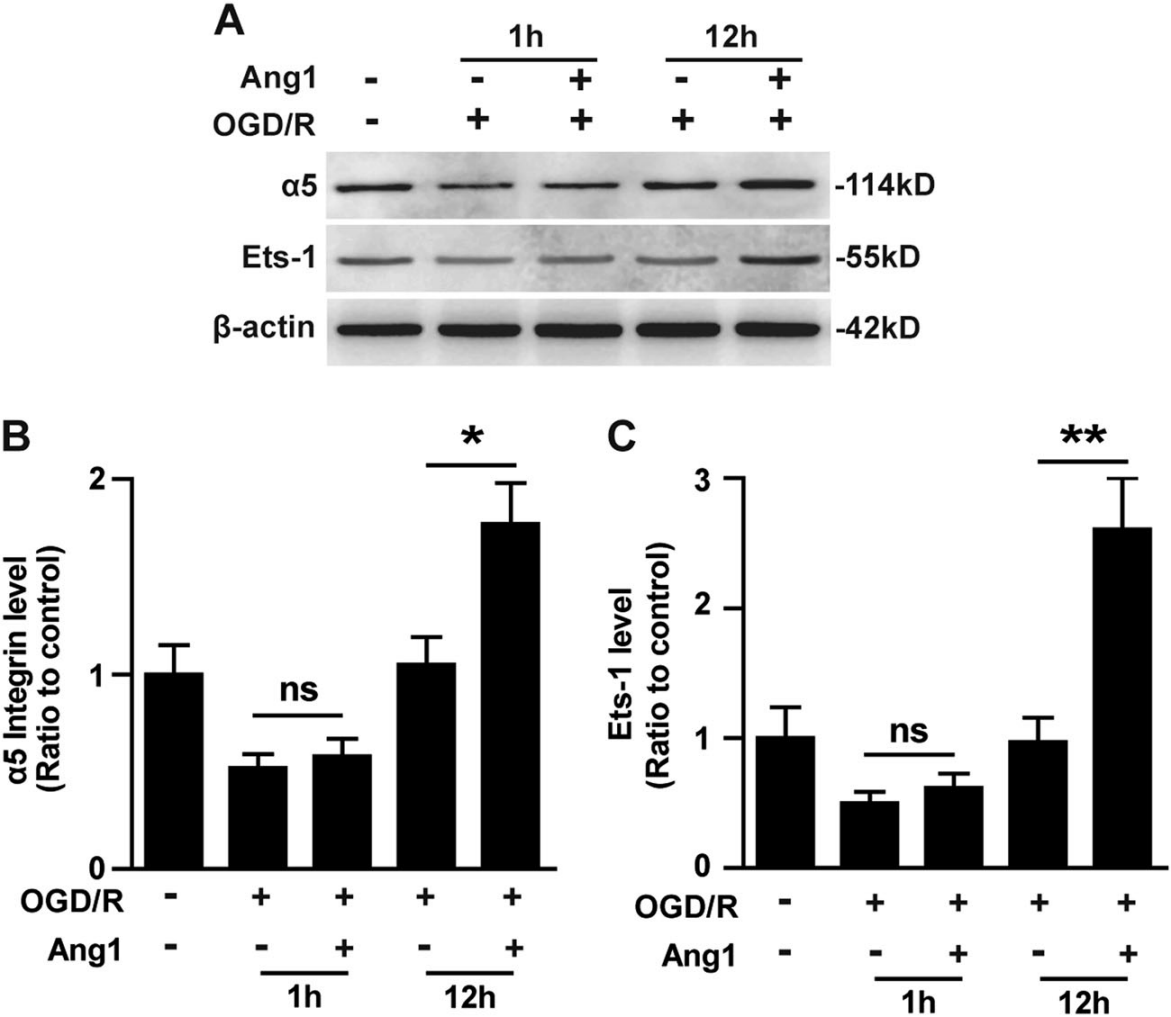
Ang1 promotes BEC expression of integrin α5 and Ets-1 under OGD/R conditions. BECs were subjected to 4 h of OGD and then stimulated or not with 100 ng/mL of Ang1 for the indicated periods under normoxia at 37 °C. **a** Cell lysates from BECs were analyzed to assess the levels of the indicated proteins by western blotting. **b**, **c** Protein levels of integrin α5 (**b**) and Ets-1 (**c**) were quantified by densitometry and are presented as ratios to β-actin. The NO-OGD cells served as the control. Data represent the mean ± SEM and were analyzed by one-way ANOVA (*n* = 3). Note that stimulation of OGD-treated BECs with Ang1 for 12 h significantly increased the expressions of both α5 and Ets-1.*P < 0.05, **P < 0.01; ns, not significant

In our recent study, we demonstrated extensive co-localization of Ang1 with α5 integrin on angiogenic blood vessels in the ischemic penumbra, suggesting that an interaction between Ang1 and ɑ5 integrin might be important in promoting angiogenesis after ischemic stroke^9^. A previous report showed that short-term stimulation of peripheral blood stem cells enriched by granulocyte colony-stimulating factor mobilization and apheresis (mobPBSCs) with COMP-Ang1 induced the expression of α5β1 integrin through Tie2 receptor downstream molecule Ets-1 signaling^11^. To examine whether a similar regulation exists in BECs under OGD conditions, we stimulated BECs with Ang1 (100 ng/mL) for 1 or 12 h immediately after OGD insult and then examined the protein levels of α5 integrin and Ets-1 by western blotting. As shown in Fig. 5a–c, stimulation of OGD-treated BECs with Ang1 for 1 h did not change the expression levels of α5 integrin or Ets-1; however, stimulation of OGD-treated BECs with Ang1 for 12 h significantly increased the expression of both α5 integrin and Ets-1 compared with the non-Ang1 treatment group (1.77 ± 0.21-fold versus 1.05 ± 0.14-fold for α5 integrin, *P* < 0.05; 2.61 ± 0.39-fold versus 0.97 ± 0.19-fold for Ets-1, *P* < 0.01) (Fig. 5b, c). These results indicate that priming of BECs with Ang1 promotes the expression of integrin α5 and Ets-1 under OGD/R conditions.

### Ets-1 is necessary for the enhanced expression of α5 integrin induced by Ang1 after OGD/R

**Fig. 6:**
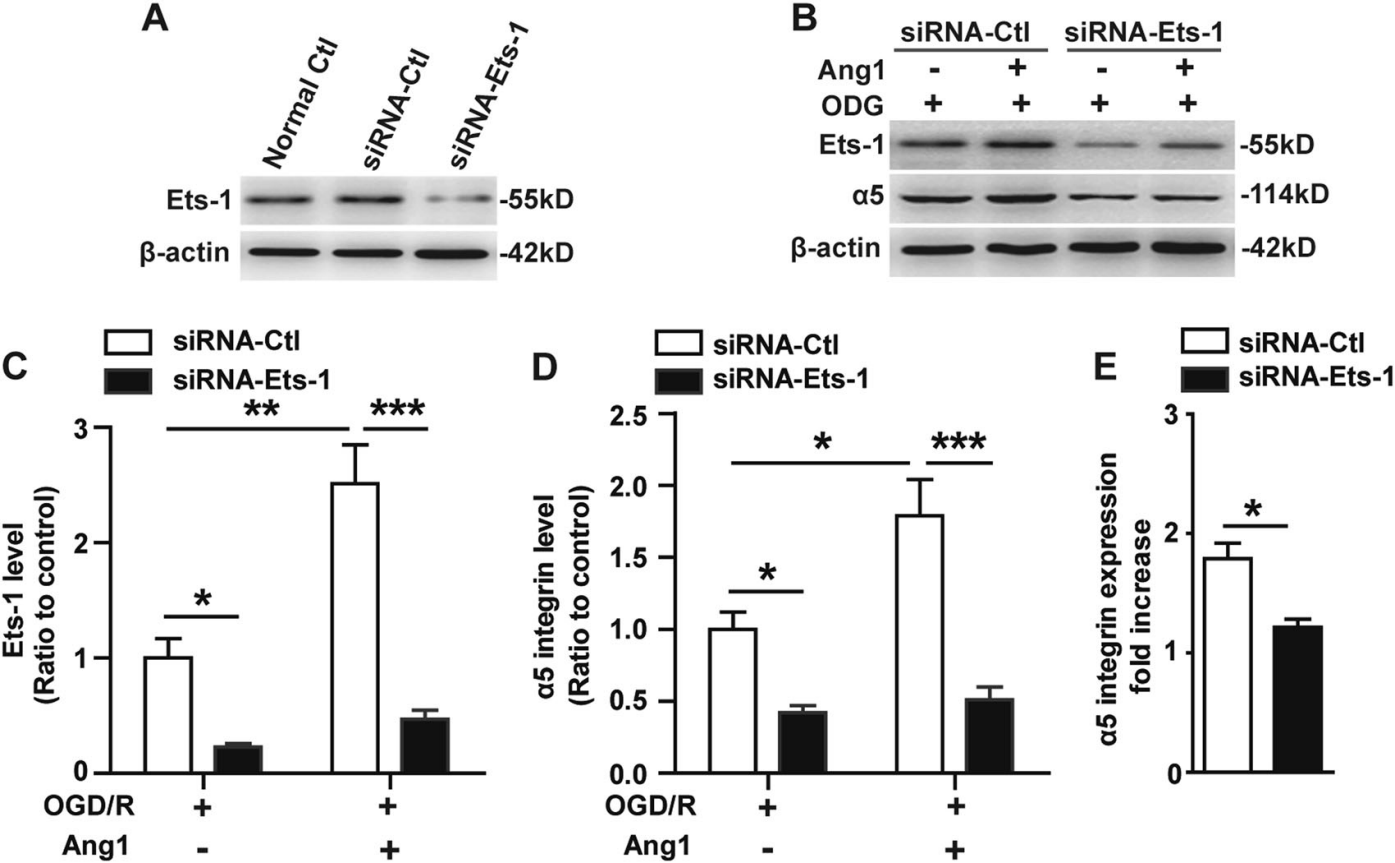
Knockdown of Ets-1 reverses the upregulation of α5 integrin induced by Ang1 after OGD. **a**. BECs were transfected with the negative control siRNA (siRNA-Ctl) or Ets-1-specific siRNA (siRNA-Ets-1) for 48 h. Levels of Ets-1 protein were monitored in the control and silenced the cells via western blotting. **b–e** BECs were transfected with siRNA-Ctl or siRNA-Ets-1 for 48 h, then subjected to 4 h of OGD, and further stimulated or not with Ang1 (100 ng/mL) for 12 h under normoxia at 37 °C. **b** The protein levels from OGD-treated BECs lysates were analyzed by western blotting using antibodies specific to integrin α5 and Ets-1. **c–e** Densitometric analysis shows the relative amount of Ets-1 (**c**) and integrin α5 (**d**) to β-actin (the OGD/R siRNA-Ctl-treated cells served as the control) and the fold increase of integrin α5 induced by Ang1 after OGD (**e**). Data represent the mean ± SEM and were analyzed by two-way ANOVA or Student’s t test (n = 3). Note that Ets-1 knockdown in BECs significantly decreased Ets-1 protein expression. Ang1 significantly upregulated integrin α5 expression on siRNA-Ctl-treated BECs relative to the OGD/R siRNA-Ctl-treated controls, but this effect was significantly reduced by Ets-1 knockdown. *P < 0.05, **P < 0.01, ***P < 0.001

Next, we examined whether the enhanced expression of α5 integrin induced by Ang1 after OGD/R is mediated by the regulation of Ets-1. BECs were transfected with the negative control siRNA (siRNA-Ctl) or Ets-1-specific siRNA (siRNA-Ets-1) for 48 h, then subjected to 4 h of OGD, and further stimulated or not with Ang1 (100 ng/mL) for 12 h under normoxia at 37 °C. As shown in Fig. 6, Ang1 significantly induced the expression of integrin α5 on siRNA-Ctl-treated BECs relative to the OGD/R siRNA-Ctl-treated controls (*P < 0.05). Furthermore, knockdown of Ets-1 expression in BECs markedly decreased integrin α5 expression in the presence or absence of Ang1 after OGD (*P < 0.05 or ***P < 0.001) (Fig. 6a–d). To directly investigate the effect of knockdown of Ets-1 on the upregulation of α5 integrin induced by Ang1 after OGD/R, we compared the difference between the fold increases of integrin α5 induced by Ang1 in siRNA-Ctl-treated controls and siRNA-Ets-1-treated BECs after OGD. This comparison revealed that diminishing the levels of Ets-1 in BECs significantly reduced the increase rate of integrin α5 induced by Ang1 after OGD compared to the siRNA-Ctl-treated controls (*P < 0.05) (Fig. 6e). These results indicate that the enhanced expression of α5 integrin induced by Ang1 after OGD/R is dependent on the upregulation of Ets-1.

### Ang1-induced BEC migration and tube formation after OGD is mediated by integrin α5

Because endothelial cell migration and tube formation constitute an important process in blood vessel formation and both the Ang1-Tie2 system^15^ and integrin α5β1^16^ are key regulators of the angiogenic process, we tested whether integrin α5β1-Ang1/Tie2 receptor cross-talk regulates the migration and tube formation of BECs.

**Fig. 7:**
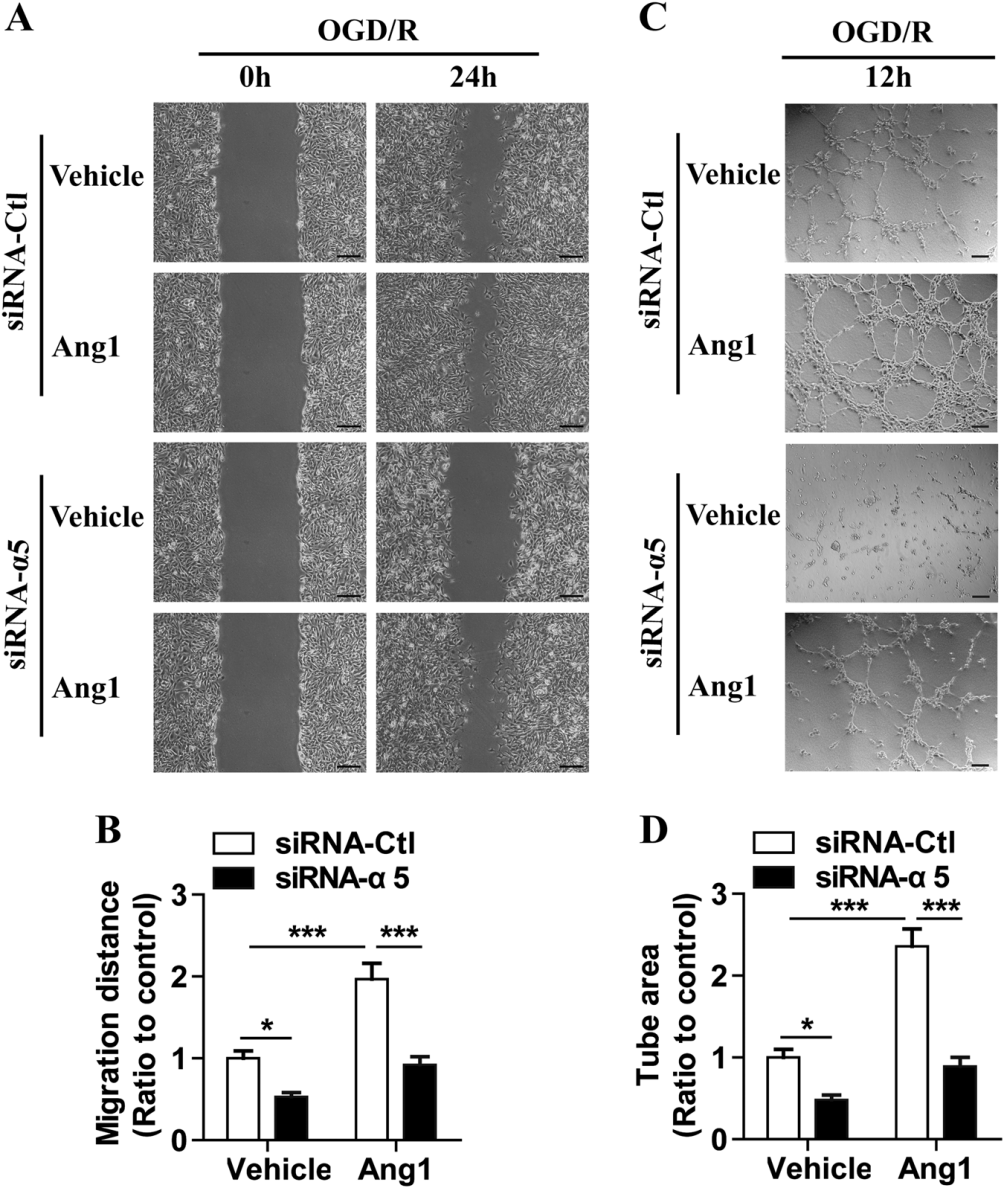
Ang1-induced BEC migration and tube formation after OGD is mediated by integrin α5. BECs were transfected with siRNA-Ctl or α5 integrin-specific siRNA (siRNA-α5), and then, cell migration and tube formation were measured after transfected BECs were subjected to 4 h of OGD. **a** Representative images of cell migration in monolayer cultured BECs in the absence or presence of Ang1 (100 ng/mL) as indicated, scale bar = 200 μm. **b** Quantification of BEC cell migration. The relative migration distance was quantified using ImageJ software and expressed as the ratio to the values of the siRNA-Ctl-treated cells (control). **c** Representative images of capillary tube formation of BECs on Matrigel-coated plates in the absence or presence of Ang1 (100 ng/mL) as indicated, scale bar = 100 μm. **d** Quantification of tube formation. Data represent the mean ± SEM and were analyzed by two-way ANOVA (*n* = 3). Note that Ang1 significantly increased the migration and capillary-like tube formation of BECs after OGD, but this effect was significantly inhibited by diminishing the levels of α5 in BECs. *P *<* 0.05, ***P < 0.001

BECs were transfected with either siRNA-Ctl or α5 integrin-specific siRNA (siRNA-α5), and then, cell migration and tube formation were measured after transfected BECs were subjected to 4 h of OGD. As illustrated in Fig. 7a, b, Ang1 treatment significantly enhanced BEC migration after 24 h of wound scratch healing relative to the siRNA-Ctl-treated controls (*P* < 0.001), but this increase was attenuated by diminishing the levels of α5 integrin in the BECs. Similarly, as demonstrated in Fig. 7c, d, considerable tube formation occurred when BECs were incubated on Matrigel for 12 h, and Ang1 strongly stimulated the BEC tube formation (*P* < 0.001) vs. the control. However, the Ang1-induced capillary tube formation was inhibited by diminishing the levels of α5 integrin in the BECs. These results indicate that the Ang1-induced migration and tube formation of BECs after OGD is mediated by integrin α5.

## Discussion

In the current study, we demonstrate for the first time that the Ang1/Tie2 system cross-talks with integrin α5β1 on BECs after experimental in vitro cerebral ischemia. Our main findings were as follows: (i) BECs subject to OGD/R showed increased expression and activation of Tie2, (ii) induction of Tie2 receptor activation by OGD/R is mediated by α5β1 integrin, (iii) priming of BECs with Ang1 promotes the expression of α5 integrin and Ets-1 after OGD, (iv) Ets-1 is necessary for Ang1 induction of α5 integrin expression on BECs after OGD/R, and (v) Ang1-induced migration and tube formation of BECs after OGD is mediated by integrin α5.

### Regulation of Ang1/Tie2 receptor signaling in BECs after OGD/R

Our recent study showed that vascular expression of both integrin α5 and Ang1 decreased during the early stages of vascular remodeling, but then increased in the later stages under cerebral ischemic conditions in vivo and in vitro^1,9^. The induction of α5β1 was first noticeable after 4 days post ischemia in vivo, followed by an increase in Ang1 at day 7 post ischemia^1,9^, which is consistent with the time course of the response to OGD/R at 24 and 48 h for α5β1 and Ang1 on BECs in vitro, respectively, indicating that the induction of Ang1 lags behind that of α5β1 after cerebral ischemia. In the current study, we found that OGD/R induced the expression of the Ang1 receptor Tie2 on BECs in a manner similar to that for integrin α5 and Ang1 in response to OGD/R, which is consistent with previous reports^17^ and our current study showing increased Tie2 expression in the ischemic border area in vivo.

We also noted that upregulation of Tie2 was accompanied by increased phosphorylation (activation) of this receptor as well as increased phosphorylation of FAK and Akt in response to OGD/R. These observations strongly suggest that OGD/R induces Ang1/Tie2 receptor signaling in BECs. As Ang1–Tie2 signaling is a key regulator of angiogenesis and adult vascular homeostasis^15^, it seems likely that enhanced Ang1/Tie2 receptor signaling will promote endogenous angiogenesis and vascular protection after cerebral ischemia.

### Integrin α5β1 regulation of cerebral ischemia-induced activation of Tie2 in BEC

In the current study, we found that α5β1 integrin co-localized with Tie2/P-Tie2 on the cerebral vessels in the ischemic penumbra after focal cerebral ischemia, showing that there is a direct interaction between α5β1 integrin and Tie2, and that α5β1 integrin might be related to the activation of Tie2 after cerebral ischemia. To confirm this, we next investigated the effect of integrin α5β1 on the activation of Tie2 in response to OGD/R. Our gene silencing experiment confirmed that diminishing BEC integrin α5β1 expression via selective shRNA-α5 resulted in the attenuation of OGD/R-induced phosphorylation of Tie2, FAK, and Akt in BECs, but the overall protein levels of Tie2 remained unaffected. The in vivo and in vitro findings suggest that integrin α5β1 is involved in the regulation of Tie2 receptor activation in BECs after cerebral ischemia. β1 integrin has previously been shown to induce phosphorylation of FAK and Akt following ligation with collagen^18^ and may thus influence the levels independently of Ang1/Tie2. However, in the current study, knockdown of α5β1 integrin in BECs did not change the phosphorylation levels of Tie2, FAK, and Akt in BECs under normal conditions. As phosphorylation of Tie2 triggered by Ang1 binding has been reported to activate specific intracellular signal molecules, including FAK, Akt, and mitogen-activated protein kinase (MAPK) (ERK1/2), in endothelial cells^12,14^, it seems that α5β1 integrin interaction with Ang1/Tie2 enhanced the activation of Tie2 and its downstream effectors FAK and Akt in BECs after cerebral ischemia. This is further confirmed by the dose response to Ang1 in both shRNA-α5 and siRNA-Ctl-treated OGD/R cells and is consistent with experiments showing that α5β1 integrin does not directly induce Tie2 signaling, but acts to sensitize Ang1-induced Tie2 activation after cerebral ischemia.

In an alternative signaling pathway, Dalton et al^14^ demonstrated that the levels of activated Akt increase in response to the Tie2 ligand Ang1, but the elevated levels of phosphorylated Akt induced by Ang1 were not significantly affected in α5 integrin knockdown telomerase-immortalized endothelial cells (TIMEs). The mechanism behind these apparent differences, from our results, may relate to the different experimental conditions, including sources of endothelial cells (Dalton used TIMEs, while our studies employed BECs). In addition, in our model, BECs were subjected to OGD/R treatment, while in the Dalton study, TIMEs were stimulated by Ang1 under normal (non-OGD) conditions. Another reason may be related to the different harvesting time points, as we examined the effect of integrin α5 knockdown on the activation of Tie2 and its downstream effectors at 48 h post-restoration, while the Dalton study monitored the changes of phosphorylated Akt just 15 min after Ang1 stimulation.

### Ang1 induction of α5 integrin expression on BECs through Ets-1 signaling after OGD

Another novel finding of our study was that priming BECs with Ang1 for 12 h promoted the expression of α5 integrin after OGD, although this phenomenon was not observed when BECs were stimulated with Ang1 for only 1 h immediately after OGD insult, presumably because it takes this long for α5 integrin protein to be upregulated at the cell surface. We also found that priming of BECs with Ang1 for 12 h induced the upregulation of Ets-1. Ets-1, a member of the v-ets erythroblastosis virus E26 oncogene homolog (ETS) transcription factors, is known to be expressed in endothelial cells during angiogenesis^19^ and be involved in the upregulation of hypoxic-inducible genes^20^. Importantly, Tie2 is reported to contain Ets-1 transcription factor binding sites in its promoter region^19^, and an Ets-1 blockade reversed the upregulation of α5 and β1 integrins in ^mob^PBSCs by COMP-Ang1. Considering these results, we postulated that elevated Ets-1 might be responsible for the upregulation of α5 integrin induced by Ang1 after OGD. As expected, the silencing experiment confirmed that knockdown of Ets-1 expression in BECs markedly decreased integrin α5 expression in the presence or absence of Ang1 after OGD. To directly investigate the effect of knockdown of Ets-1 on the upregulation of α5 integrin induced by Ang1 after OGD/R, we compared the difference between the fold increases of integrin α5 induced by Ang1 in siRNA-Ctl-treated controls and siRNA-Ets-1-treated BECs. This comparison revealed that diminishment of the levels of Ets-1 in BECs significantly reduced the increase rate of integrin α5 induced by Ang1 after OGD compared to the siRNA-Ctl-treated controls. These results suggest that the enhanced expression of α5 integrin induced by Ang1 after OGD/R is dependent on the upregulation of Ets-1.

### Integrin α5 regulation of Ang1-induced migration and tube formation of BECs after OGD

The Ang1–Tie2–Akt system is believed to be a key regulator of several angiogenic processes, including endothelial cell survival, migration, sprouting, and tube formation^15^. The expression of α5β1 integrin is upregulated in response to angiogenic factors^21^, and α5β1 integrin has previously been shown to be involved in mediating cell migration and tube formation in human choroidal endothelial cells^16^. In this study, we further found that Ang1 induces cell migration and tube formation of BECs after OGD, but this effect was attenuated by diminishing the levels of α5 integrin in BECs. Similarly, our previous study and other reports showed that antagonists of α5β1 integrin blocked tumor necrosis factor-α, bFGF, and interleukin-8-stimulated angiogenesis, but had a minimal effect on the vascular endothelial growth factor (VEGF)-A response^22,23^. This evidence indicates that Ang1/Tie2 elicits angiogenesis after cerebral ischemia through strict cooperation with α5β1 integrin. VEGF has also previously been shown to activate Tie receptors^24^, thus VEGF might be an alternative target to influence Tie2 activation independently of α5β1 integrin. Therefore, further studies specifically designed to investigate the effects of VEGF and its interrelationship with α5β1 integrin on Tie2 activation after cerebral ischemia should be done before a conclusion can be reached.

**Fig. 8:**
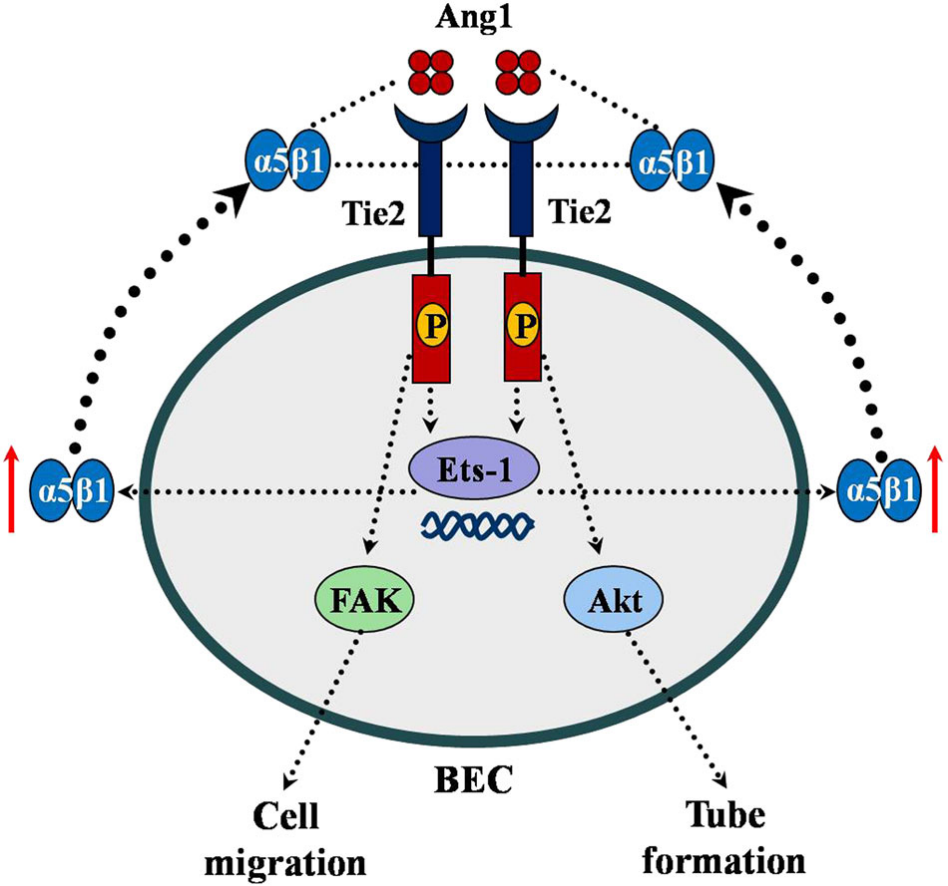
Schematic representation of Ang1/Tie2 and integrin α5 cooperative signaling in brain endothelial cell regulation. Following OGD, Ang1/Tie2 is upregulated, which cooperates with integrin α5 to induce Tie2 activation and the phosphorylation of its downstream signaling molecules FAK and Akt in BECs, resulting in the increase of migration and capillary-like tube formation of BECs. Additionally, priming of BECs with Ang1 promotes the expression of α5 integrin through Ets-1 signaling after OGD. Abbreviations: angiopoietin-1, Ang1; brain endothelial cells, BECs; v-ets erythroblastosis virus E26 oncogene homolog-1, Ets-1; focal adhesion kinase, FAK; phosphorylation, P

In summary, our results demonstrate that the Ang1/Tie2 system interacts with integrin α5β1 on BECs after cerebral ischemia. Specifically, integrin α5β1 regulates cerebral ischemia-induced BEC activation of Tie2 and priming of BECs with Ang1 via the Tie2/Ets-1 pathway, significantly upregulating the expression of integrin α5. Functionally, integrin α5 regulates Ang1-induced migration and tube formation of BECs after OGD (Fig. 8). Knowledge of these molecular and signaling pathways within BECs may facilitate the design of drugs aimed at promoting angiogenic remodeling immediately after cerebral ischemia and thus contribute to novel vascular protective strategies in the treatment of ischemic stroke.

## Electronic supplementary material

Additional Figure Legends

Additional figure 1
