# Supplementary material for: Integrin α5β1-Ang1/Tie2 receptor cross-talk regulates brain endothelial cell responses following cerebral ischemia
Source: Exp Mol Med. 2018 Sep 5;50(9):117. doi: 10.1038/s12276-018-0145-7 (PMC6123805; doi:10.1038/s12276-018-0145-7)
Supplement: Supplementary file 1 — Additional Figure Legends [file 12276_2018_145_MOESM1_ESM.docx]

**Figure legend for additional figure 1**

OGD/R induces upregulation of integrin α5 expression in BECs

BECs were subject to 4 hours of oxygen glucose deprivation (OGD) followed by 72 hours of restoration (R) at 37°C. Cell lysates at each time point were analyzed by Western blotting using antibodies specific for integrin α5 (#4705, Cell Signaling Technology, Inc.). A. Representative Western blots of integrin α5 in BEC are shown. B. Protein levels were quantified by densitometry and presented as ratios to β-actin. Note that OGD/R induced a marked increase in BEC expression of α5 integrin, with the maximum level of expression for this antigen reached 48–72 h after restoration. The result for integrin α5 antibody (#4705, Cell Signaling Technology, Inc.) gives identical results to integrin α5 antibody (AB1928, Millipore) used in our current study. Data represent mean±SEM and analyzed by one-way ANOVA (n=5). ***P*< 0.01, ****P*< 0.001 compared with NO-OGD/R control.
