# Supplementary figures and images for: Integrin α5β1-Ang1/Tie2 receptor cross-talk regulates brain endothelial cell responses following cerebral ischemia
Source: Exp Mol Med. 2018 Sep 5;50(9):117. doi: 10.1038/s12276-018-0145-7 (PMC6123805; doi:10.1038/s12276-018-0145-7)

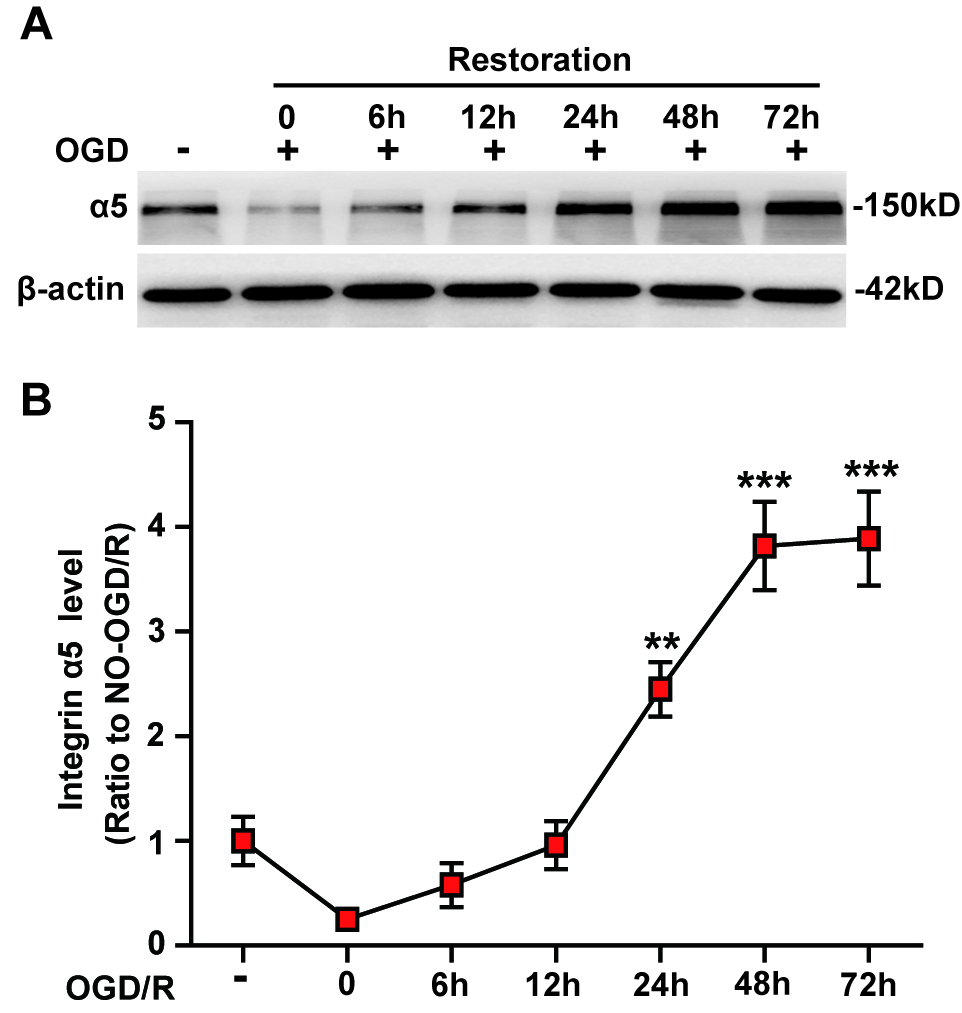

Supplement: Supplementary file 2 — Additional figure 1 [file 12276_2018_145_MOESM2_ESM.tif]
